# A novel miniaturized filamentous phagemid as a gene delivery vehicle to target mammalian cells

**DOI:** 10.1016/j.omtn.2025.102571

**Published:** 2025-05-19

**Authors:** Shirley Wong, Salma Jimenez, Deborah Pushparajah, Rohini Prakash, Roderick Slavcev

**Affiliations:** 1 School of Pharmacy, University of Waterloo, Waterloo, ON N2L 3G1, Canada; 2 Centre for Eye and Vision Research (CEVR), 17W, Hong Kong Science Park, Science Park, Hong Kong

**Keywords:** MT: Delivery Strategies, filamentous bacteriophage M13, phagemid, gene transfer, non-viral gene delivery, DNA minivector

## Abstract

The filamentous phage M13 is a single-stranded DNA phage with several attractive characteristics for gene delivery, including a capsid amenable to the display of foreign peptides and a simple well-characterized genome that is easy to genetically modify. Previously, we constructed a DNA minivector based on M13 (a miniphagemid), which minimized the inflammatory bacterial and phage DNA content in the vector. In general, DNA minivectors devoid of their prokaryotic components have shown improved gene transfer and safety. We examined the miniphagemid’s capacity for *in vitro* transgene delivery to target cells through phage display of epidermal growth factor to target its cognate receptor. The absence of the prokaryotic backbone and smaller vector size conferred by the miniphagemids were associated with improved transgene expression for purified single-stranded phagemid DNA and phagemid virion particles. We further engineered this system to enhance packaging of DNA minivectors via deletion of the packaging signal within the helper plasmid used to produce miniphagemids and observed improved phage-mediated gene expression in mammalian cells. Overall, we present a set of novel transgene delivery vectors that combine cell-targeting ligand display and vector minimization. This platform showcases the flexibility of M13 as a gene delivery tool with immense therapeutic potential.

## Graphical abstract

**Figure:**
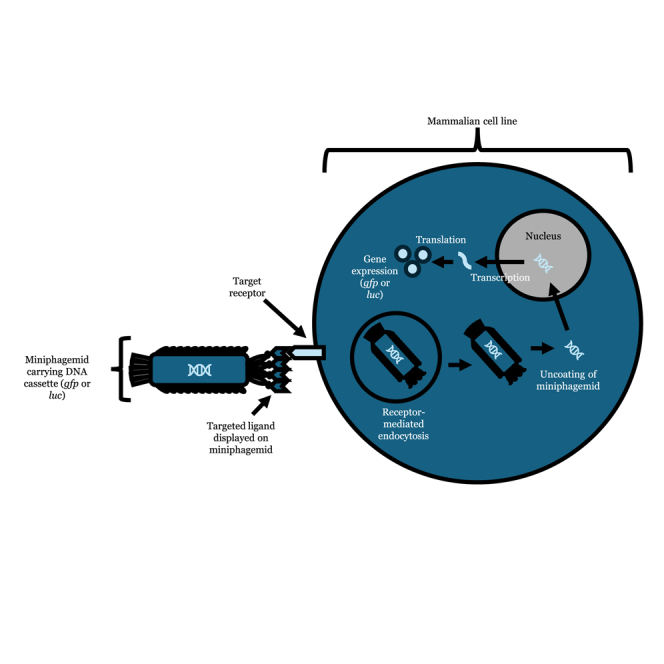

## Teaser

This study investigates the use of engineered M13 bacteriophage as a gene delivery vector for mammalian cells. By displaying an EGF cell-targeting ligand and eliminating prokaryotic elements, M13 miniphagemids were shown to improve gene delivery and expression. Additionally, this study optimizes packaging to enhance the efficiency and safety of M13 miniphagemids for gene delivery applications.

## Introduction

Viruses, such as the filamentous bacteriophage (phage) M13, exist as inert proteinaceous particles outside their host bacterium, *Escherichia coli (E. coli)*. As they do not possess intrinsic tropism for mammalian cells, they can function as simplistic carriers of genetic material for gene transfer applications. However, phages also have no intrinsic means by which to transduce mammalian cells. Indeed, the most common gene transfer vectors are modified viruses of mammalian and human origin^1^^,^^2^ and exhibit natural tropism for human cells. However, some safety and efficacy concerns limit their usage, specifically, high immunogenicity, risk of insertional mutagenesis, and the possibility of recombination into replication-competent viruses.^3^^,^^4^^,^^5^^,^^6^^,^^7^ In contrast, non-viral methods of gene transfer incorporate other means of bypassing cellular barriers for gene transfer, such as with chemical transfection reagents that mediate transport across cell membranes.^8^^,^^9^ However, these transfection reagents cannot target specific tissues; functionalization requires additional covalent linkage which may not be amenable to scale-up.^8^

As phages do not innately enter nor replicate within mammalian cells, they may be considered as “non-viral” gene transfer vectors. To overcome their lack of tropism, display of a cell-targeting ligand on a phage capsid can facilitate cellular uptake by exploiting receptor-mediated endocytosis.^10^^,^^11^^,^^12^ Genetic incorporation of a sequence for phage display easily facilitates decoration of the phage capsid with any ligand of choice. The filamentous phage M13 is an excellent model for the display of cell-specific ligands. The phage’s simplistic genome encodes all proteins necessary for replication, progeny assembly, and extrusion, which are controlled by signaling structures within the phage replicative origin (f1 *ori*) in the genome. The f1 *ori* contains all sequences necessary and sufficient to direct phage-mediated replication of a DNA molecule. Therefore, any conventional plasmid that also contains an f1 *ori*, in addition to its plasmid *ori*, can be replicated by filamentous phage machinery independent of its plasmid origin^13^^,^^14^ and assembled into virion particles. Phage particle length is dependent on the size of the encapsulated DNA, so, theoretically, there is no limit to the transgene capacity of M13. In practice, the physical limits still encompass a large range: “microphage” variants carrying as little as 221 nt of DNA^15^^,^^16^ to “polyphage” particles carrying as much as ten phage genome copies (over 60 kilonucleotides [knt] of DNA) have been observed.^17^

The prokaryotic backbone is necessary for amplification and maintenance in a bacterial host; however, it is rich in unmethylated cytosine-guanine dinucleotide (CpG) motifs that are known to inhibit transgene expression in mammalian cells.^18^ Indeed, CpG-mediated immunostimulation constitutes a major part of the immune response against M13 administered in mammals.^19^^,^^20^ The prokaryotic backbone of a typical plasmid used for gene delivery furthermore contains an antibiotic resistance marker that can disseminate antibiotic resistance into the environment.^21^ Ultimately, this backbone does not contribute at all to transfection and is thus unnecessary bulk. We and others have documented efforts in producing precursor vectors for M13-mediated production of DNA minivectors (Figure S1) deficient in bacterial or phage genetic sequences.^22^^,^^23^ The precursor vector contains a split M13 origin enabling packing of the gene of interest including its expression elements producing miniphagemids, while full phagemids contain the whole precursor plasmid (Figure S2). Here, we evaluated if filamentous M13 miniphagemid particles can improve phage-mediated delivery of a mammalian transgene cassette (*cmv-luc*) based on the display of a cell-specific ligand for internalization and the absence of the prokaryotic backbone. We also further engineered vectors for M13-mediated production to improve DNA minivector packaging by limiting the packaging of full precursor DNA and helper plasmid in order to investigate if this could consequently enhance gene delivery and expression in mammalian cells.

## Results

### Display of a cell-specific ligand

To determine the effect of displaying the ligand of interest, epidermal growth factor (EGF), on phage titers, M13SW7-EGF titers were measured by plaque assays (Table S1), which were comparable to those of its predecessors, M13SW7 and M13KO7, as well as wild-type M13. This indicates that the EGF fusion was well tolerated by the phage and did not impede infectivity. No significant difference was observed in packaging efficiency by phage displaying or not displaying EGF (Figure S4). Instead, packaging efficiency was largely determined by the phagemid. Miniaturized phagemids were more preferentially packaged over their full phagemid counterparts.

**Figure 1:**
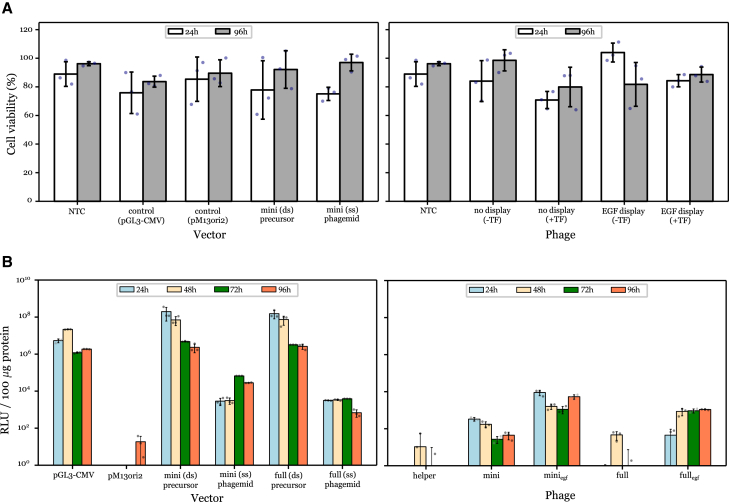
Cell viability is unaffected while transgene expression increases after vector administration Purified DNA (left) and phage (right) were administered to HeLa cells (A) Cell viability was assessed through the MTT assay 24 h and 96 h post-transfection, while (B) gene expression was assessed through a luciferase assay. Raw luminescence of firefly luciferase was reported 24, 48, 72, and 96 h after transfection. The plasmid precursor pM13ori2.cmvluc (mini precursor) and the corresponding purified ssDNA of mini-(luc) (miniphagemid) were transfected alongside the source plasmid pGL3-CMV and the empty vector pM13ori2 as controls. Helper phage M13KO7 (no display) and M13SW7-EGF (EGF display) were transfected alone or with TurboFect. Purified dsDNA was transfected at 1 μg/mL, ssDNA at 2 μg/mL, and phage particles at 5 × 10^7^ virions/mL. NTC, no treatment control; TF, TurboFect. Error bars represent SD, *n* = 3. The dots represent individual values of the replicates.

M13 phage particles also did not adversely impact mammalian cell viability. This was regardless of EGF display or complexation with a commercial cationic polymer (TurboFect) both at 24 h and 96 h after transfection (Figure 1A). Phage particles did not reduce cell viability to any significant degree in comparison with the delivery of purified DNA. Purified plasmid double-stranded DNA (dsDNA) and phagemid single-stranded DNA (ssDNA) were transfected over a period of 96 h in HeLa cells (Figure 1B), using the empty backbone vector pM13ori2 as a negative control and pGL3-CMV (the source of the *cmv-luc* cassette) as a positive control. Luciferase activity peaked approximately 24–48 h post-transfection, and no significant difference was observed between any of the three dsDNA plasmid vectors. Overall, gene expression, as measured by luciferase activity, was approximately 100-fold lower for purified ssDNA compared to each plasmid counterpart, with expression peaking approximately 24 h later, at 72 h. Notably, transfection of purified mini-(*luc*) ssDNA was correlated with increased luciferase activity over purified full-(*luc*) ssDNA. Similar trends were observed when transfecting intact phage particles. Display of EGF dramatically increased gene expression for both mini and full phagemid vectors, which underlines the necessity of receptor-mediated cell internalization for phage-mediated gene transfer.

**Figure 2:**
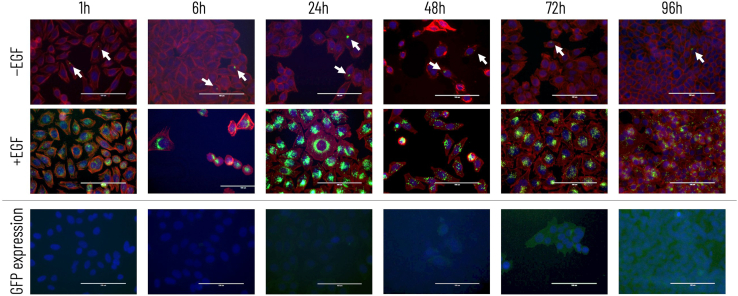
Localization of phage over time in HeLa M13KO7 (−EGF) and M13SW7-EGF (+EGF) were tagged with Alexa Fluor 488 (green; top two rows) and applied to HeLa cells and then visualized between 1 and 96 h. Cell nuclei were stained with DAPI (blue), and the cytoskeleton was stained with rhodium phalloidin (red). In the bottom row, GFP expression (green) was visualized after administration of mini_egf_-(*gfp*) phage. Nuclei were stained with DAPI. The scale bar size is indicated in each image as 100 nm.

To further characterize the necessity of a targeting ligand, HeLa cells were treated with EGF^+^ or EGF^−^ phage for 1, 6, 24, 48, 72, and 96 h. The use of EGF as a cell-targeting ligand has previously been shown to improve gene transfer by filamentous phage both *in vitro* and *in vivo*.^11^^,^^12^^,^^24^^,^^25^ Upon ligand binding, internalized epidermal growth factor receptor (EGFR)-EGF complexes are routed to lysosomes for degradation; therefore, EGFR-bound phage may be prone to accumulate within juxtanuclear lysosomes, which positions them perfectly for subsequent escape and nuclear transport. Alexa Fluor-tagged M13SW7-EGF abundantly localized to HeLa cells within an hour of administration in a ligand-dependent manner, while M13KO7 did not associate with HeLa cells in any appreciable levels (Figure 2). After 6 h, EGF-displaying phage surrounded the nucleus, indicating successful cell uptake and cytoplasmic translocation. It is known that EGF-EGFR complexes can internalize within 15–20 min,^26^ while receptor-bound phages can internalize as early as 10–60 min after administration.^27^^,^^28^^,^^29^^,^^30^ Our results appear consistent with these findings.

### Expression efficiency of miniphagemid-mediated gene delivery

**Figure 3:**
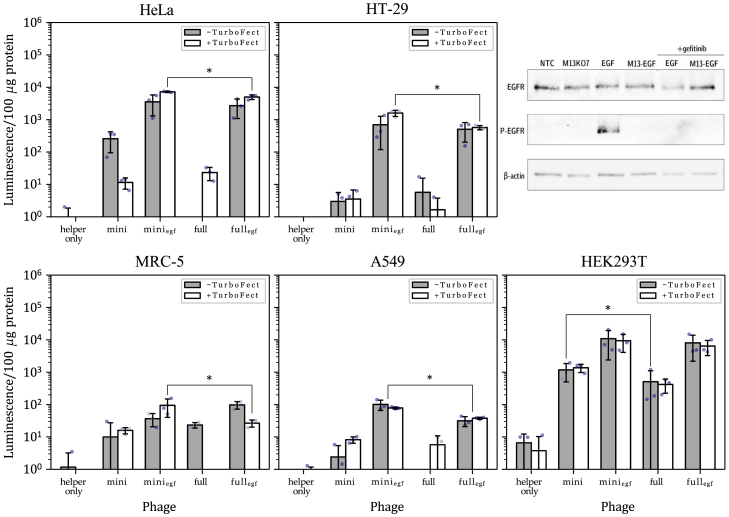
Miniphagemids confer improved transgene expression across EGFR^+^ cell lines Transgene expression was assessed by luciferase assay conducted 96 h after administration of M13SW7 mini or full phagemids encoding *cmv-luc*. Error bars represent SD, *n* = 3. The ∗ above the bars indicates a difference at significance level *p* < 0.05. The dots represent individual values of the replicates. EGFR phosphorylation was characterized in HeLa cell extracts (western blot, top-right), after administration of M13KO7 (no display), purified recombinant EGF (100 ng/μL), or M13SW7-EGF (EGF display) for 5 min prior to analysis. Additionally, cells were pre-treated with the EGFR inhibitor, gefitinib (10 μM), prior to treatment with EGF or M13SW7-EGF. Cell lysates were probed for the presence of EGFR (EGFR) or phosphorylated EGFR (P-EGFR). Β-actin was used as the loading control.

The capacity of M13SW7 mini and full phagemids for gene transfer was then compared across four cell lines from different tissues known to moderately express EGFR^31^^,^^32^^,^^33^^,^^34^^,^^35^^,^^36^: HeLa, HT-29, MRC-5, and A549, as well as an EGFR^−^ cell line, HEK293T.^31^^,^^37^^,^^38^ Firstly, it can be noted that miniphagemids confer increased gene expression compared to full phagemids as an approximately 3-fold increase was observed for most of the cell lines tested (Table 1). In EGFR^+^ cells, the biggest factor contributing to increased gene expression was the display of the cell-specific ligand (Table 2; Figure 3). This is expected, as the phage must first be taken up through receptor-ligand interactions before any benefits from improved cytoplasmic trafficking can manifest. Although the display of the receptor-targeting ligand was necessary for gene expression, it alone was insufficient for high levels of transgene expression. The rate of phage internalization clearly relies on a number of different factors relating to both the phage used and the targeted tissue type.^27^ Fold differences in luciferase activity between EGF-displaying and non-displaying counterparts showed a dramatic increase in gene expression from the display of EGF (Table 2; Figure 3), indicating a requirement for receptor-mediated vector internalization when the receptor was present. Gene expression was maximally 700-fold greater in HeLa cells and over 100-fold greater in HT-29 cells when the miniphagemid displayed EGF. Overall, our results are generally consistent with previous reports of EGF-mediated improved phage internalization and subsequent gene transfer.^24^^,^^25^^,^^39^

**Table 1:** Fold difference in luciferase expression between M13SW7 mini and full phagemids, *n* = 3

Cell line	Fold difference in gene expression (mini/full)			
	EGF^−^		EGF^+^	
	−TurboFect	+TurboFect	−TurboFect	+TurboFect
HEK293T	5.22 ± 5.63	4.21 ± 3.79	2.03 ± 2.59	1.78 ± 1.79
HeLa	∞^a^	0.51 ± 0.12	1.98 ± 1.92	1.48 ± 0.37
HT-29	∞^a^	7.44 ± 7.63	1.74 ± 1.40	2.81 ± 0.67
MRC-5	0.52 ± 1.04	∞^a^	0.43 ± 0.33	3.51 ± 1.73
A549	∞^a^	∞^a^	3.20 ± 0.05	2.08 ± 0.18

^a^∞: luminescence below threshold for full phagemid.

**Table 2:** Fold difference in luciferase expression between M13SW7-EGF-displaying and non-displaying phage, *n* = 3

Cell line	Fold difference in gene expression (EGF^+^/EGF^−^)			
	Mini		Full	
	−TurboFect	+TurboFect	−TurboFect	+TurboFect
HEK293T	11.73 ± 11.07	7.17 ± 4.24	43.94 ± 59.44	17.65 ± 10.54
HeLa	25.59 ± 33.01	704.94 ± 351.36	∞^a^	261.37 ± 182.45
HT-29	∞^a^	∞^a^	∞^a^	∞^a^
MRC-5	∞^a^	5.73 ± 2.79	4.14 ± 0.47	∞^a^
A549	∞^a^	9.96 ± 3.34	∞^a^	∞^a^

^a^∞: luminescence below threshold for EGF^−^ phagemid.

**Figure 4:**
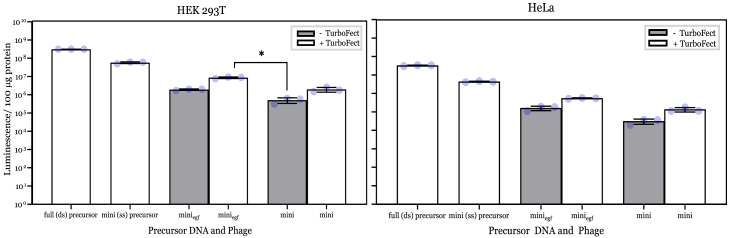
M13SW8-packaged miniphagemids confer improved transgene expression across mammalian cell lines Transgene expression was assessed by luciferase assay 96 h after treatment of M13SW8 miniphagemids encoding *cmv-luc* to HEK293T and HeLa cells. Error bars represent SD, *n* = 3. The ∗ above the bars indicates a difference at significance level *p* < 0.05. The dots represent individual values of the replicates.

Considering that miniphagemids conferred higher gene expression, we next examined M13SW8 and M13SW8-EGF miniphagemids, packaged with mini-(*luc*) ssDNA, for gene expression efficiency in HEK293T (EGFR^−^) and HeLa cells (EGFR^+^). We previously demonstrated that the loss of the packaging signal (PS) in the helper phage genome of M13SW8 reduced helper self-packaging, thereby increasing the proportion of miniphagemids carrying the transgene cassette of interest within the miniphagemid lysate.^22^ Lysates of both M13SW8 and M13SW8-EGF miniphagemids showed negligible levels of contaminating phagemid particles, indicating little to no contaminating helper phage or full precursor DNA (Figure S5; Table 3). An increase in gene expression was observed when treated with EGF-displaying M13SW8 miniphagemids in comparison to non-EGF-displaying M13SW8 miniphagemids (Figure 4) at 96 h, as observed for M13SW7 (Figure 3). Fold differences in gene expression between M13SW8 and M13SW7 showed a range of approximately 40- to 2,000-fold increase in HeLa and HEK293T cells (Table 4) for EGF-displaying and non-EGF-displaying miniphagemids. Overall, these results demonstrate increased levels of gene delivery when using M13SW8 as compared to M13SW7, suggesting that improvements in the packaging efficiency of DNA minivectors promote increased gene delivery and expression.

**Table 3:** Efficiency of plating (EOP) for M13SW8 and M13SW8-EGF miniphagemids

Miniphagemid	Total titer (particle/mL)	Contaminated helper phage EOP^a^	Contaminated full phagemid EOP^b^	Total EOP^c^
M13SW8-mini-(luc)	5.73 × 10^13^	<1.74 × 10^−11^	<3.50 × 10^−11^	<1.74 × 10^−11^
M13SW8-mini_egf_-(luc)	3.32 × 10^12^	<3.01 × 10^−10^	<3.01 × 10^−10^	<3.01 × 10^−10^

a Contaminated helper phage EOP calculation: contaminated helper phage titer/total phagemid titer.

b Contaminated full precursor phagemid EOP calculation: contaminated full precursor phagemid titer/total phagemid titer.

c Total EOP calculation: total contaminated phagemid (helper phage + full precursor phagemid) titer/total phagemid titer.

**Table 4:** Fold differences in gene expression between M13SW8 and M13SW7 miniphagemids, *n* = 3

Cell line	Fold difference in gene expression (M13SW8/M13SW7)			
	EGF^+^		EGF^-^	
	-TurboFect	+TurboFect	-TurboFect	+TurboFect
HEK293T	270.08 ± 21.25	570.88 ± 45.65	788.11 ± 215.99	2131.78 ± 499.77
HeLa	40.08 ± 9.00	79.03 ± 3.63	4742.63 ± 1194.37	1939.45 ± 421.40

### Filamentous phage uptake is independent of EGFR phosphorylation

Despite the use of EGFR as an internalization target in multiple studies, activation of EGFR-mediated signal transduction by EGF-displaying phage has not been deeply investigated. Canonically, ligand-EGFR interactions lead to receptor dimerization and receptor autophosphorylation prior to internalization of the entire receptor-ligand complex via clathrin-coated endocytosis.^40^^,^^41^ Activation of the EGF receptor stimulates signal transduction pathways involved in cell proliferation. Phosphorylation of several intracellular tyrosine residues of EGFR mediates downstream signal transduction. After treatment of HeLa cells with purified recombinant EGF, phosphorylated EGFR was detectable but not if cells were pre-treated with gefitinib, an EGFR inhibitor (Figure 3). In contrast, no phosphorylation was detected after treatment with EGF-displaying phage. The M13 phage particle itself appears sufficient to prohibit EGFR ligand-activated autophosphorylation, without preventing ligand-activated internalization.

## Discussion

Enhancing gene delivery has been widely explored for various studies using different physical, chemical, and biological methods.^42^ Exploration of using phage as gene delivery vehicles are included in these studies, including M13 phage,^22^^,^^30^^,^^43^ which was investigated in this study. We generated miniphagemids carrying minimal gene cassettes while also displaying a targeting ligand, EGF. Through this, we were able to visualize miniphagemid localization in mammalian cells, in addition to measuring levels of gene expression *in vitro* to assess the efficiency of miniphagemid gene delivery.

We previously demonstrated improved miniphagemid rescue efficiency via deletion of the PS in the helper phage genome,^22^ which we additionally confirmed here. Significant packaging of the helper genome is a common issue with helper phages such as M13SW7 and M13KO7. Phage genomes in the resultant lysate add contaminating, potentially immunogenic bacterial DNA and reduces the concentration of transgene-encoding molecules per phage lysate. Consequently, the system was optimized by deleting the PS of M13KO7, generating M13SW8, in order to prevent packaging of self-DNA.^22^ Miniphagemids produced using M13SW8 would therefore be expected to exhibit enhanced gene delivery and expression as a higher percentage of target miniphagemids should be produced. When HEK293T and HeLa cells were transfected with M13SW8 miniphagemids, an increase in luciferase expression compared to M13SW7 miniphagemids was observed, indicating that the use of a self-packaging-deficient helper phage resulted in superior lysates for transfections. This is most likely due to an increased amount per volume of target miniphagemids and a decreased amount of non-targeting phagemid bulk delivered to target cells.

To produce phagemids with EGF display, this peptide was fused to pIII, as mentioned earlier. As pIII is a minor coat protein and does not participate in phagemid replication or ssDNA sequestration, it was not expected to impact miniphagemid production^22^^,^^25^. This was shown as quantification of the phage lysates showed that the fusion did not inhibit helper phage rescue of phagemid from either a split or wild-type origin. Since the display fusion was encoded on the helper phage, other fusion peptides could be incorporated without modifying the phagemid vector itself. For chimeric display on other coat proteins (e.g., pVIII), it may be possible to incorporate a fusion peptide on the backbone of the phagemid vector such that it would not be assembled into progeny viral particles. Additionally, in future studies it would be ideal to replace the EGF-targeting ligand with other targeting ligands and determine the effect of this on phage production, in addition to the targeting of different cell types.

Localization of the produced phagemids demonstrated the strong perinuclear accumulation of tagged phage particles, which suggests that they did not yet escaped the endosomal compartment at 6 h, as escaped phage particles are expected to localize more diffusely throughout the cytoplasm.^30^ Still, expression of phage-encoded transgene cassettes was detectable 48–72 h post-treatment, which was on par with the delivery of purified ssDNA. Either the additional requirement of DNA separation from the filamentous phage coat does not significantly delay phage-mediated gene transfer or potential improvements from phage-mediated cell uptake and intracellular navigation are sufficient to offset delays. Indeed, the low luminal pH of juxtanuclear lysosomes can contribute to phage coat shedding,^44^ possibly improving the bioavailability of phage-encapsulated DNA.

Considering that removal of the bacterial backbone conferred an increase in gene expression as compared to the full M13SW7 phagemids with an intact bacterial backbone, it can be suggested that mini DNA vectors conferred enhanced gene delivery. This increase in gene expression was statistically significant in combination with the display of EGF and when complexed with TurboFect in all four EGFR^+^ cell lines (*p* < 0.05) for M13SW7 phagemids, which supports this suggestion. However, in HEK293T cells, a cell line with low EGFR expression, phagemid miniaturization correlated with increased luciferase activity when complexed with TurboFect (*p* < 0.05), but there was no statistically significant difference between phagemids with or without ligand display. For M13SW8 miniphagemids transfected in HEK293T cells, statistical significance (*p* < 0.05) was observed for miniphagemids displaying EGF and complexed with TurboFect, compared to non-displaying miniphagemids without complexing with TurboFect. This suggests that phage uptake may have occurred independently of the targeting ligand and, thus, the potential for nonspecific tissue uptake. However, this contradicts the observed inability of EGF^–^ phage to transfect some of the EGFR^+^ cell lines tested. It may be that HEK293T cells express a cell surface receptor with intrinsic affinity for M13; however, studies of M13 in mice have not shown strong preferential accumulation in the kidney.^45^ Filamentous phage particles have been previously reported to enter via caveola-mediated endocytosis,^46^^,^^47^ while larger phage particles enter via phagocytosis and micropinocytosis.^48^ In the absence of a target receptor, filamentous phage uptake in HEK293T is likely clathrin independent, but the specific endocytic mechanism and how the phage targets the cell remain unclear and require further investigation.

Intriguingly, phage-mediated gene expression was slightly improved when combined with TurboFect, a cationic polymer, which is consistent with the observations of Donnelly et al., who reported that the cationic polymer polyethylenimine (PEI) improved filamentous phage-mediated gene transfer.^49^ Complexation with a cationic polymer enhanced EGF-dependent gene transfer but did not permit EGF-independent gene transfer, suggesting that its benefits may be realized intracellularly. This is more indicative of a role in facilitating endosomal escape rather than cell entry. In future studies, it would be ideal to test miniphagemid gene delivery with different types of formulations including lipid nanoparticle delivery systems and assess its influences.

Larger molecules, such as the monoclonal antibody cetuximab^50^ and bacteriophage λ,^51^ have been shown to internalize through EGFR binding without triggering the downstream signaling pathway.^52^ Antagonistic receptor binding by cetuximab and λ have been shown to reduce cell proliferation. However, we and others have not observed a decrease in cell viability over time after administration of EGF^+^ filamentous phage. Intriguingly, the induction of late EGFR-stimulated events has been reported with other EGF-displaying M13: specifically, M13 display of EGF was involved in the activation of c-fos serum response element-mediated transcription.^53^^,^^54^ Still, it has been demonstrated elsewhere that these events can occur even in cells with kinase-defective EGFR,^55^^,^^56^ suggesting alternative mechanisms to stimulate EGFR-mediated signal transduction separately from the EGF receptor specifically. Downstream cell proliferation pathways could be activated in the absence of cell surface EGFR phosphorylation.^57^^,^^58^^,^^59^ While we did not observe phage-mediated EGFR autophosphorylation, this does not rule out endosomal EGFR signaling nor other kinase-independent signal transduction. Further investigations with EGF-displaying phage are warranted. Overall, both purified and phage-encapsulated miniphagemid DNA were correlated with greater gene expression over their full phagemid counterparts, which we attributed to the reduction in immunostimulatory CpG motifs and improved cytoplasmic diffusion. Another benefit conferred by the smaller size of the phagemid particle may be more efficient internalization upon ligand-receptor binding. Clathrin-mediated endocytosis has been previously shown to accommodate filamentous phage particles up to 900 nm in length,^30^ even though clathrin-coated vesicles are typically on the scale of 200 nm^60^ Although long, their flexible rod-like structure enables filamentous phages to be compacted; as such, they are more readily taken up alongside receptor-mediated clathrin or caveola-mediated endocytosis in contrast to other larger, more globular proteins and phages. This effect is likely enhanced by the shorter nature of the miniphagemid particles since filamentous phage length is determined largely by the length of the encapsulated DNA molecule.

In general, it should be noted that a limitation of the studies conducted to assess *in vitro* gene delivery was the use of only luciferase expression levels, opposed to additionally using GFP, which could have provided transfection efficiency measures using flow cytometry. GFP is often less sensitive for reporting gene expression compared to luciferase^61^ and therefore, likely contributed to some of the dim levels of visible GFP when viewing for phage localization via fluorescence microscopy. Additionally, luciferase requires less time for detection of expression promoting faster data retrieval at measurable levels.^62^ Future work will expand on different reporters for fully characterizing intracellular localization and gene delivery of internalized phage particles.

In this study, we demonstrated the enormous potential of the filamentous phage M13 for *in vitro* gene delivery. Previous studies show its ability to display a variety of foreign peptides and its ability to be easily genetically modified due to its simple genome.^22^^,^^30^^,^^43^ We showed that highly pure lysates of miniphagemids encapsulating DNA minivectors led to enhanced expression levels in mammalian cells in a ligand-dependent manner, highlighting their potential to be used as a safe, targeted, and efficient gene delivery vector for the treatment of a variety of diseases. These could include genetic disorders, cancer, infectious diseases, and more. Before approaching this stage, *in vivo* studies in mouse models are required to test the safety, immune response (inflammatory markers), and delivery efficiency of miniphagemids. Overall, the results obtained from this study shows the potential use of M13 miniphagemids as an effective gene delivery vehicle with minimized side effects due to the absence of a prokaryotic backbone within the DNA vector(s) it encapsulates, and efficiency in manufacturing as miniphagemids are easy to produce in high quantities and demonstrated high levels of purity.

## Materials and methods

### Strains and vectors

*E. coli* K-12 JM109 was used in the generation of all phage and plasmid constructs. All bacterial and mammalian cell lines are listed in Table S2, plasmids in Table S3, and phages in Table S4. All mammalian cell lines were maintained in tissue culture plates (Thermo Scientific) at 37°C in a humidified atmosphere with 10% CO_2_ and cultured in Dulbecco’s modified Eagle’s medium (Thermo Scientific) supplemented with 10% heat-inactivated fetal bovine serum and 1% penicillin/streptomycin. Bacterial strains were cultured in Luria-Bertani (LB) liquid medium, supplemented with the relevant antibiotic as required. The list in Table S3 includes the plasmids that were tested for packaging within the produced full and miniphagemids, in addition to helper plasmids which were constructed as outlined in the subsequent section. The list in Table S4 outlines the full and miniphagemids that were produced in this study. Phages were purified through precipitation with polyethylene glycol (PEG) and stored in Tris-NaCl (TN) buffer as previously described.^22^

### Construction of M13KO7 derivatives with pIII::EGF fusion display

M13KE has endonuclease target sites in gIII (KpnI-EagI) that simplify N-terminal peptide fusions,^63^ while M13KO7 contains the plasmid p15a *ori* for phage-independent amplification and a kanamycin resistance marker (KanR) marker for antibiotic selection.^64^ The *gIII* from M13KE was inserted into the helper phage M13KO7 using Gibson assembly to generate the helper phage M13SW7, which can easily take on N-terminal pIII fusions while retaining the p15a *ori* to simplify selection and amplification. Primers are summarized in Table S5. Next, the peptide EGF was inserted as a pIII N-terminal fusion with a GGGS (Gly-Gly-Gly-Ser) linker^65^ between the KpnI-EagI sites of M13SW7. Insertion was verified in the final construct (M13SW7-EGF) through PCR of the phage lysate (Figure S3A). Peptide display of EGF was verified through dot blot and ELISA using anti-EGF antibodies (Thermo Fisher Scientific) (Figures S3B and S3C). An additional self-packaging-deficient variant of M13KO7 (M13SW8) was engineered with a deleted PS and pIII:EGF fusion display, as previously described.^22^ Display of the EGF peptide was completed in the same manner as M13SW7-EGF, to generate M13SW8-EGF. Purification of phage, double-stranded DNA, and single-stranded miniphagemid DNA was completed as previously described, in addition to quantification of phage lysates.^22^^,^^66^

### Assessment of M13SW8 packaging efficiency

Spot plates of M13SW8-mini-(luc) and M13SW8-mini_egf_-(luc) were conducted to assess for packaging contamination of helper plasmids (M13SW8 and M13SW8-EGF) and full precursor plasmid (pM13ori2.cmvluc) by evaluating the ability of the produced phagemids to confer antibiotic resistance to susceptible cells. As pM13ori2.cmvluc contains an ampicillin resistance (AmpR) marker and the helper phage plasmids (M13SW8 and M13SW8-EGF) each contain a KanR marker, any infected colony growth on ampicillin, kanamycin, and ampicillin + kanamycin LB agar plates demonstrates packaging contamination since miniphagemids should only package the gene cassette of interest. To conduct the spot plate technique, 300 μL of *E. coli* JM109 cells was added to 3 mL of top agar supplemented with 5 mM MgSO_4_ and poured onto pre-warmed LB plates with relevant antibiotics (ampicillin, kanamycin, and ampicillin + kanamycin). 10 μL of miniphagemids, diluted in TN buffer from 10^−2^ to 10^−8^, were plated. The plates were incubated overnight at 37°C. Efficiency of plating (EOP) was then calculated by the following formulas: contaminated helper phage titer/total phagemid titer, contaminated full precursor phagemid titer/total phagemid titer, and total contaminated phagemid (helper phage + full precursor phagemid) titer/total phagemid titer.

### Assessment of cell viability after exposure to phage particles

HeLa cells were seeded in 96-well plates (Thermo Fisher Scientific) at 5 × 10^4^ cells/mL. The following day, EGF-displaying (M13SW7-EGF) and non-displaying (M13KO7) phage were transfected into each well at concentrations between 5 × 10^7^ virions/mL. Cell viability was assayed using 3-(4,5-dimethylthiazol-2-yl)-2,5-diphenyltetrazolium bromide (MTT) after 24 and 96 h by measuring A_492_ on a Varioskan LUX multimode plate reader (Thermo Fisher Scientific). Cell viability was reported as a percentage of the difference between the absorbances of each sample (A_sample_) and negative control (A_negative_) relative to the absorbance of the untreated control, A_NTC_: (A_sample_ – A_negative_)/A_NTC_.

### Localization of transfected phage particles

PEG-precipitated M13SW7-EGF (EGF^+^) and M13KO7 (EGF^−^) were labeled with Alexa Fluor 488 (Thermo Fisher Scientific). HeLa cells were seeded in 24-well plates at 5 × 10^4^ cells/mL. The following day, labeled phage particles and mini-(*gfp*) were transfected into cells at 5 × 10^7^ virions/mL. Wells were imaged 1, 6, 24, 48, 72, and 96 h after transfection. To image, cells were first fixed with 4% para-formaldehyde and permeabilized with 0.1% Triton X-100. Nuclei were stained with 4′,6-diamidino-2-phenylindole (DAPI), while actin was stained with rhodamine phalloidin. Fixed cells were imaged on the EVOS FL auto imaging system (Thermo Fisher Scientific) at 40× magnification using the DAPI (nuclei), red fluorescent protein (actin), and GFP (phage or expressed GFP) channels.

### Transfection of phage in EGFR+ cell lines

Cell lines were seeded at the following cell densities in 24-well plates: 5 × 10^4^ cells/mL (HeLa) and 1 × 10^5^ cells/mL (HEK293T, MRC-5, and HT-29). Phage particles were added to a final concentration of 5 × 10^7^ virions/mL of phagemid. Helper phage alone was transfected as a negative control. To assess the influence of a cationic polymer transfection carrier, phage particles were also complexed with 2 μL of TurboFect. Transfection was quantified through luminescence of firefly luciferase expression (Luciferase Reporter Assay System; Promega) after 96 h. Luminescence was normalized against whole protein content, which was estimated via a bicinchoninic acid assay (Thermo Fisher Scientific). The efficiency of gene transfer was reported as luminescence per 100 μg of whole protein content (relative light units [RLU]/100 μg).

### Phosphorylation of EGFR in HeLa

Activation of EGFR, as assessed by phosphorylation of the tyrosine residue at 1,173, was visualized via western blot. HeLa cells were treated with EGF^−^ phage (M13KO7), recombinant EGF (Thermo Fisher Scientific), or EGF^+^ phage (M13SW7-EGF) for 5 min prior to cell lysis. This was repeated in cells pre-treated with EGFR inhibitor gefitinib (Cell Signaling Technology) 2 h prior to addition of EGF or M13SW7-EGF. The western blot was performed using anti-EGF antibodies as outlined earlier, including the use of β-actin as a loading control. Lysates were probed for phosphorylated EGFR using a phospho-specific EGFR rabbit monoclonal antibodyspecifically against Tyr1173 (53A5, Cell Signaling Technology).

### Statistical analysis

Values are reported as means of *n* independent experiments with uncertainty reported as the standard deviation (SD). Statistical hypothesis tests were evaluated using one-way ANOVA, followed by the Tukey range test for multiple comparisons. Values of *p* < 0.05 were considered statistically significant. The phagemid fraction was determined as the concentration of target phagemid divided by the total virion concentration, expressed as percentages. As compositional data,^67^ they have a fixed constant sum constraint (100%). In order not to violate this constraint, the data were transformed using an isometric log ratio transformation before performing statistical analyses and transformed back to percentages for reporting.

## Data availability

All relevant data are within the manuscript.

## Acknowledgments

The authors offer many thanks to J. Blay, M. Aucoin, and N. Oviedo for their generosity in sharing materials. This work was supported in part by the Natural Sciences and Engineering Research Council of Canada (grant number 391457); New Frontiers in Research Fund (NFRF); 10.13039/501100004489Mitacs Canada and 10.13039/501100013704CONACYT Mexico; and the Centre for Eye and Vision Research (CEVR), InnoHK, and HKSAR

## Author contributions

Conceptualization, S.W., S.J., and R.S.; methodology, S.W., S.J., and R.S.; formal analysis, S.W.; investigation, S.W., S.J., D.P., and R.P.; visualization, S.W.; writing – original draft preparation, S.W.; writing – review and editing, S.J., R.S., D.P., and R.P.; supervision, R.S.; funding acquisition, R.S.

## Declaration of interests

S.W. and R.S. have submitted a patent application from the work reported in this manuscript (United States Provisional Patent Application No. 63/336,844).

## Supplemental information

Document S1. Figures S1–S5 and Tables S1–S5

Document S2. Article plus supplemental information
