# Supplementary material for: A novel miniaturized filamentous phagemid as a gene delivery vehicle to target mammalian cells
Source: Mol Ther Nucleic Acids. 2025 May 19;36(2):102571. doi: 10.1016/j.omtn.2025.102571 (PMC12173652; doi:10.1016/j.omtn.2025.102571)
Supplement: Document S1. Figures S1–S5 and Tables S1–S5 [file mmc1.pdf]

OMTN, Volume *36*

Supplemental information

A novel miniaturized filamentous phagemid as a gene delivery vehicle to target mammalian cells

Shirley Wong, Salma Jimenez, Deborah Pushparajah, Rohini Prakash, and Roderick Slavcev

Table S1: Phage titres

Phage	Titre (x 10^{13} PFU/mL)
M13	0.13 ± 0.05
M13KO7	1.90 ± 0.10
M13KE	0.12 ± 0.02
M13SW7	3.43 ± 1.50
M13SW7-EGF	2.75 ± 0.90

Table S2: Strains used in this study

Strain	Genotype/description	Source
**Bacterial strains**		
JM109	F′ *traD36 proAB+ lacIq lacZΔM15/ Δ(lac-proAB) endA1 glnV44 thi-1 e14− recA1 gyrA96 relA1 hsdR17*	New England BioLabs
**Mammalian cell lines**		
HEK-293T	Embryonic kidney, epithelial	Gift, Dr. M. Aucoin
HeLa	Uterus, cervix adenocarcinoma	Gift, Serenity Bioworks
MRC-5	Lung, fibroblast	ATCC: CCL-171
HT-29	Colon, epithelial adenocarcinoma	Gift, Dr. J. Blay
A549	Lung, epithelial carcinoma	ATCC: CCL-185

Table S3: Plasmids used in this study

Plasmid	Genotype	Source
pGL2-SS-CMV-GFP-SS	pGL2-Promoter, *cmv-gfp* replaces SV40-*luc*, AmpR	Gift, Mediphage Bioceuticals
pGL3-CMV	pGL3-Basic, *cmv* inserted in BlgII-HindIII, AmpR	Gift, Dr. N. Oviedo
pBluescript II KS+	Wild-type f1 *ori*, pUC *ori*, AmpR	[68]
M13SW8	M13KO7, Packaging signal removed, Kn^R	Our previous study[22]
M13SW8-EGF	M13SW8, EGF display on pIII, Kn^R	This study
M13SW7	M13KO7, gIII from M13KE	This study
M13SW7-EGF	M13SW7, *egf* inserted in KpnI-EagI	This study
pSW9	pBluescript II KS+, *cmv-gfp* inserted in KpnI, AmpR	Our previous study[22]
pSW10	pBluescript II KS+, *cmv-luc* inserted in KpnI, AmpR	Our previous study[22]
pM13ori2	pUC57, M13-START and M13-STOP in *lacZa*	Our previous study[22]
pM13ori2.cmvgfp	pM13ori2, *cmv-gfp* from pGL2-SS-CMV-GFP-SS inserted in EcoRI-PacI	Our previous study[22]
pM13ori2.cmvluc	pM13ori2, pM13ori2, *cmv-luc* from pGL3-CMV inserted in EcoRI-KpnI	Our previous study[22]

Table S4: Phages used in this study

Phage	Genotype	Source
M13KO7	Tn903 (p15a *ori*, KanR)	New England BioLabs
M13KE	lacZα, KpnI & EagI in gIII	New England BioLabs
M13SW7	M13KO7, gIII from M13KE	This study
M13SW7-EGF	M13SW7, *egf* inserted in KpnI-EagI	This study
M13SW7-full-(gfp)	from precursor pSW9, AmpR	This study
M13SW7-full$_{egf}$-(gfp)	from precursor pSW9, AmpR	This study
M13SW7-full-(luc)	from precursor pSW10, AmpR	This study
M13SW7-full$_{egf}$-(luc)	from precursor pSW10, AmpR	This study
M13SW7-mini-(gfp)	from precursor pM13ori2.cmvgfp, AmpR	This study
M13SW7-mini$_{egf}$-(gfp)	from precursor pM13ori2.cmvgfp, AmpR	This study
M13SW7-mini-(luc)	from precursor pM13ori2.cmvluc, AmpR	This study
M13SW7-mini$_{egf}$-(luc)	from precursor pM13ori2.cmvluc, AmpR	This study
M13SW8-mini-(luc)	from precursor pM13ori2.cmvluc, AmpR	This study

Table S5: Primers for M13SW7 and M13SW7-EGF phage construction

Primer	Amplicon	Sequence (5′ − 3′)
gIII-F	gIII	TTTTTTGGAGATTTTCAACGTGAAAAAATTATTATTCGCAATTCC
gIII-R	gIII	CCCAAAAGAACTGGCATGATTTAAGACTCCTTATTACGCAGTATG
M13KO7-F	M13KO7	GTTGAAAATCTCCAAAAAAAAGGC
M13KO7-R	M13KO7	TCATGCCAGTTCTTTTGGG
KpnI-egf-F	*egf*	*GGTACCTTTCTATTCT*CACTCTAATAGTGACTCTGAATGTC CC[b]
EagI-egf-R	*egf*	CGGCCGA*AGAACCACCACC*GCGCAGTTCCCACCAC[c]

[a]Underlined nucleotides indicate primer overhang. [b]Italicized nucleotides indicate gIII leader sequence.
[c]Italicized nucleotides indicate GGGS linker.

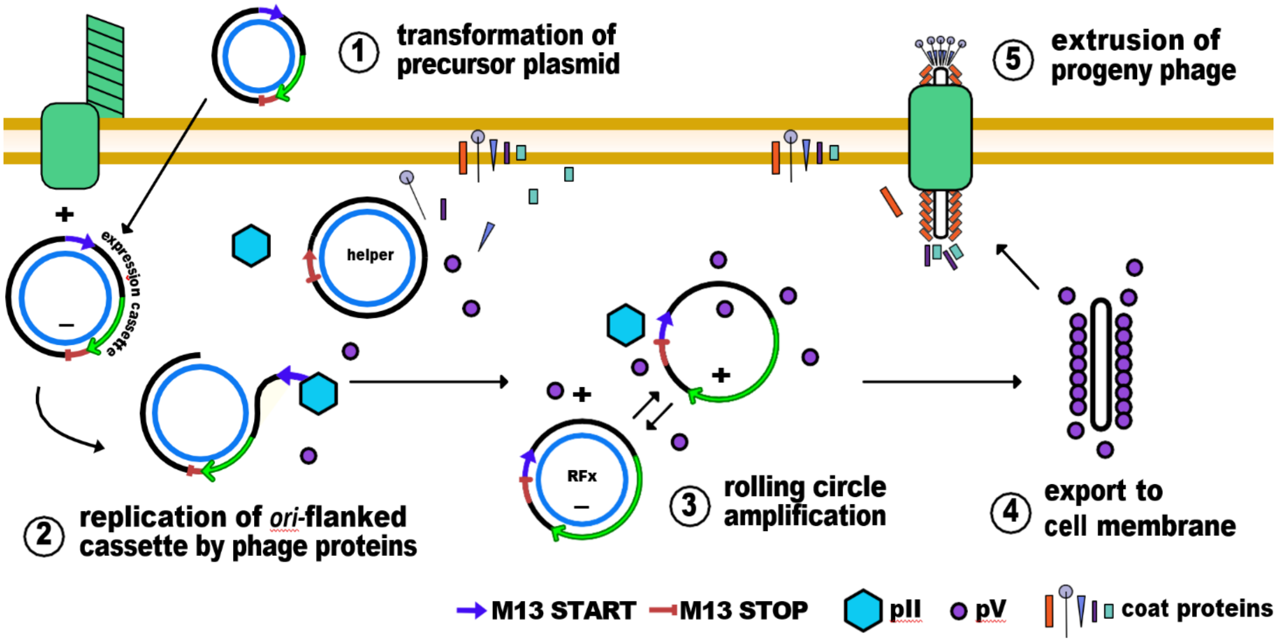

Figure S1: Production and purification of miniphagemid particles. 1) The host cell is transformed by the precursor plasmid encoding a region of interest between the separated M13 replication signals. 2) After infection with a helper phage encoding a display peptide, phage protein expression can occur. 3) Rolling circle amplification generates a recombinant replicative factor (RFx) from the plasmid that reconstitutes the f1 *ori* and loses the plasmid backbone. 4) The phage ssDNA binding proteins pV sequester single-stranded minivector, preventing further amplification, and shift the replication cycle towards assembly. 5) Assembly proteins extrude progeny phage particles encapsulating the minivector that homogeneously display a peptide of interest.[22]

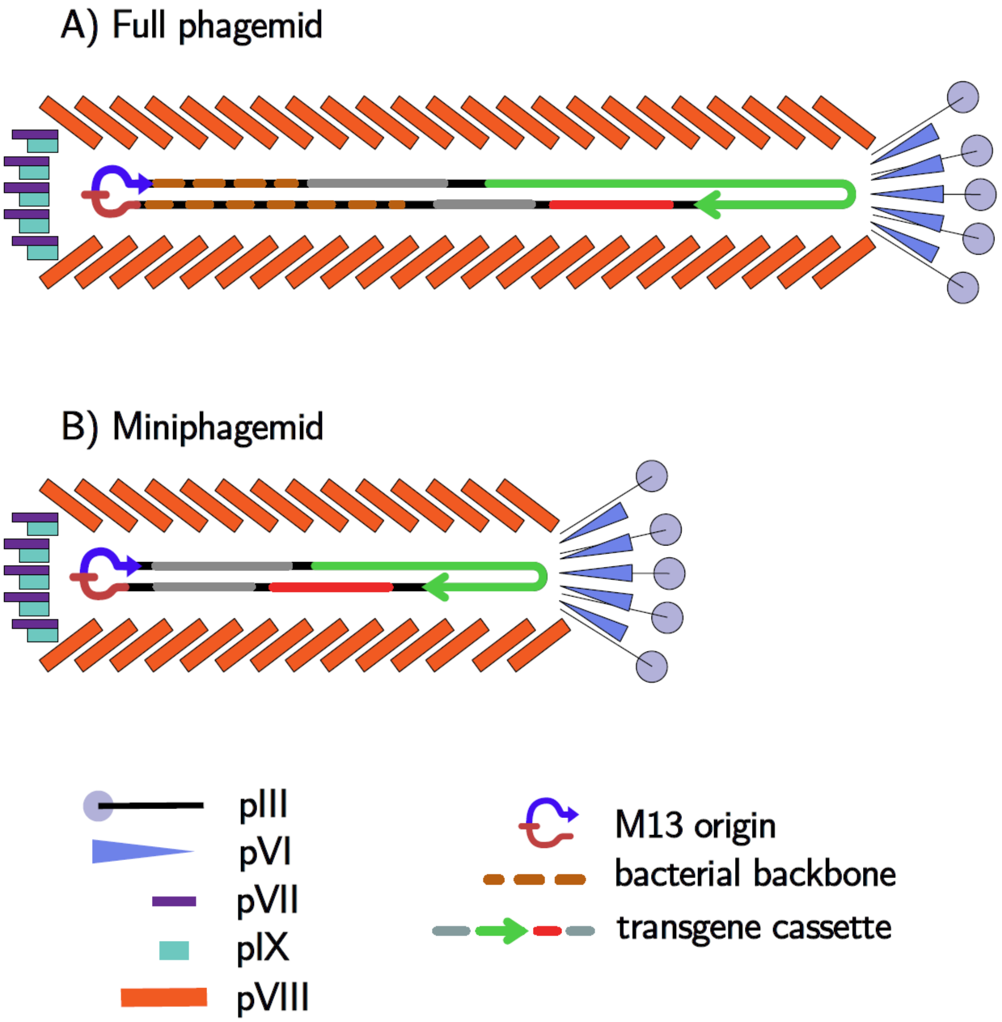

Figure S2: Full phagemid verses miniphagemid diagram A) Full phagemids encapsulate the full precursor plasmid including the M13 origin, bacterial backbone and transgene cassette. B) Miniphagemids encapsulate only the transgene cassette including the M13 origin, eliminating the presence of the bacterial backbone.

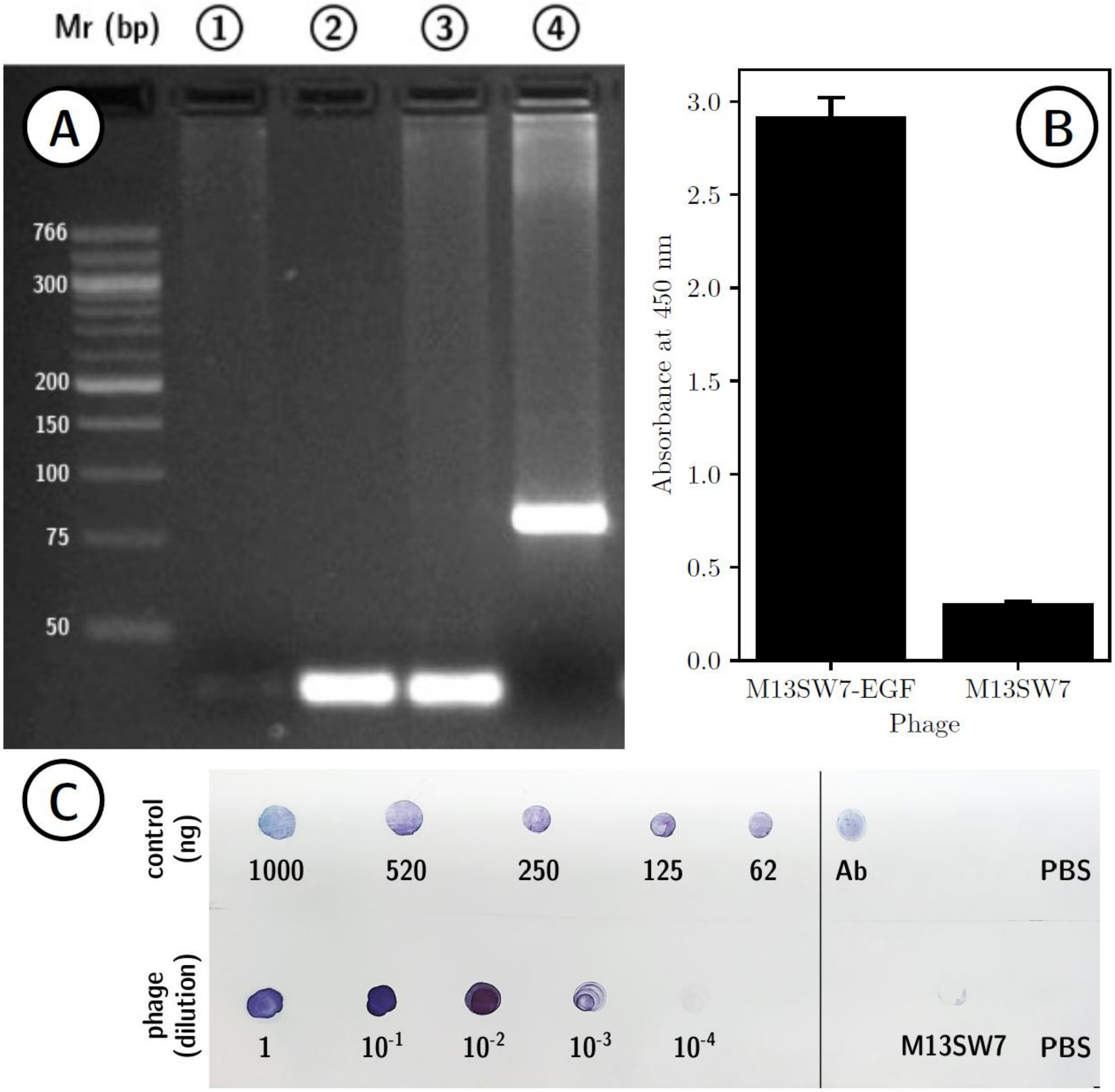

Figure S3: EGF display is confirmed on recombinant helper phage. A) The KpnI-EagI region in *gIII* was amplified by PCR and visualized via AGE. From left to right: M13KO7 (negative control, no KpnI-EagI region), M13KE and M13SW7 (no EGF; 54 bp KpnI-EagI region), 4) M13SW7-EGF (228 bp), Mr: Low Molecular Weight Ladder (New England BioLabs). B) ELISA of the putative EGF^{+} helper M13SW7-EGF is compared to the EGF^{-} precursor, M13KO7. Error bars represent SD, *n*=3. C) A dot blot comparing recombinant EGF (control, top) to M13SW7-EGF (bottom); controls: antibody spotted directly on the membrane (positive), EGF^{-} phage (negative) and phosphate-buffered saline (negative).

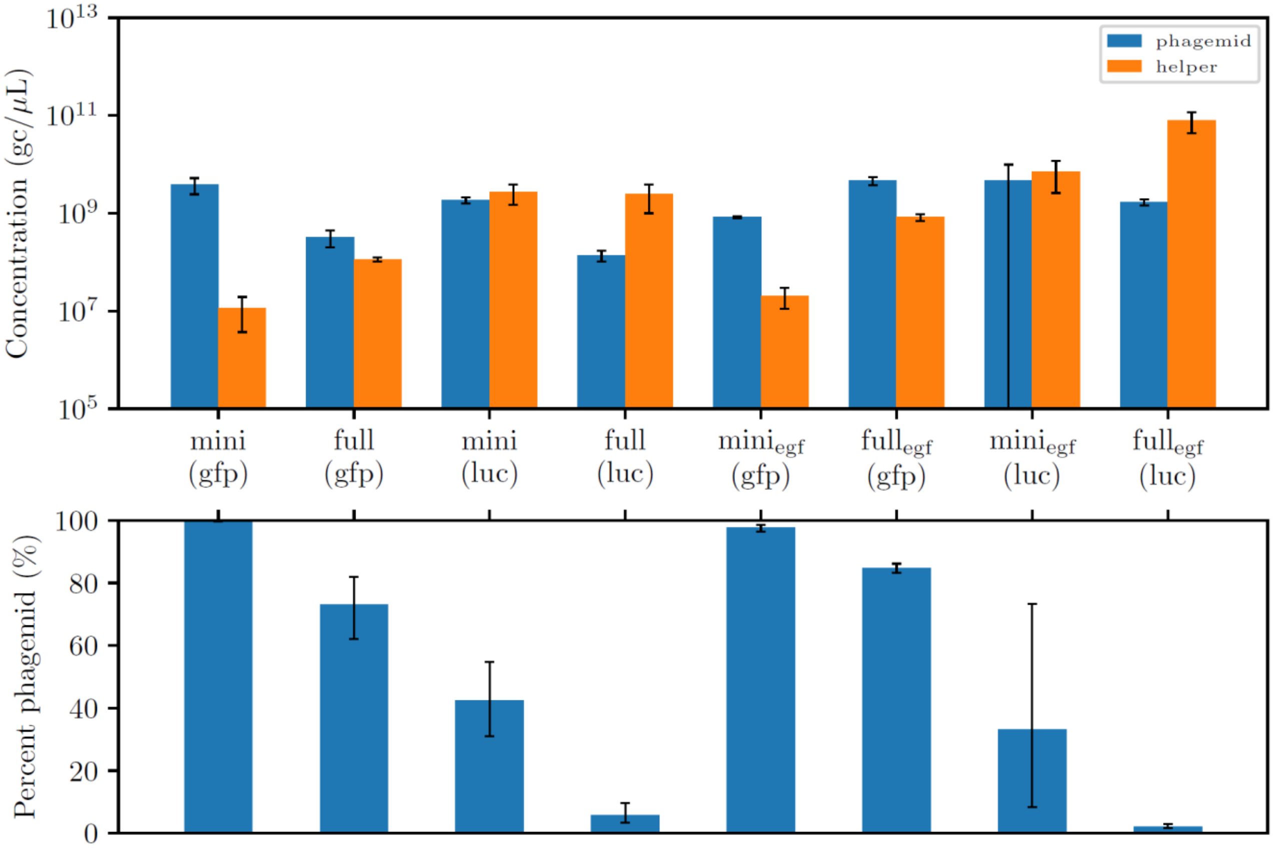

Figure S4: Composition of EGF-displaying phage lysates. At the top, the concentration (gc/µL) of each phage species (target phagemid or helper) in each lysate is presented. Below, the phagemid fraction of each phage species is shown as a percentage of the total phage population. Phagemids on the left were rescued by helper phage M13KO7 (EGF^-), while phagemids on the right were rescued by helper phage M13SW7-EGF (EGF^+). Error bars represent SD, *n*=3.

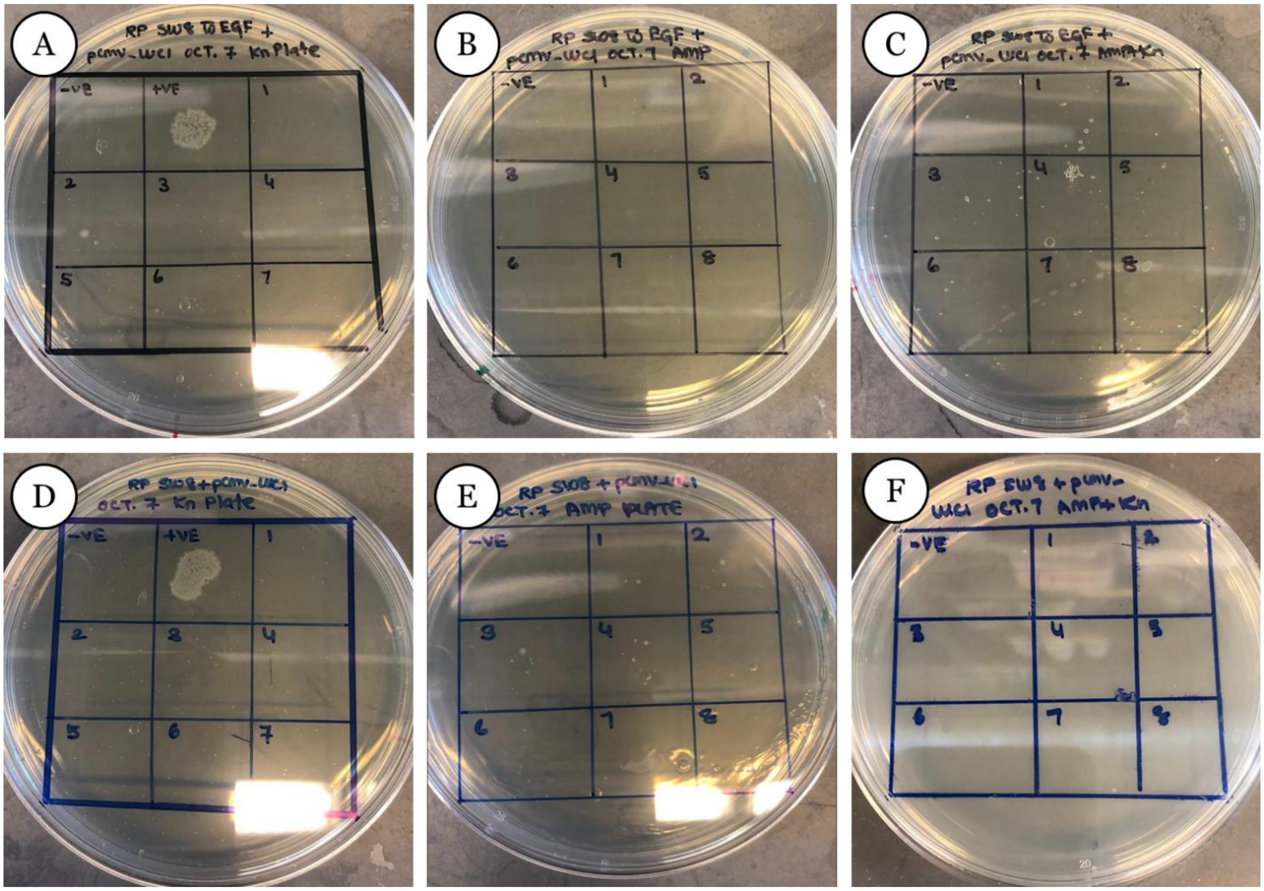

Figure S5: Efficiency of plating spot plates. M13SW8-mini$_{egf}$-(luc) spot plates on a) LB + kanamycin; b) LB + ampicillin; c) LB + ampicillin + kanamycin. M13SW8-mini-(luc) spot plates on d) LB + kanamycin; e) LB + ampicillin; f) LB + ampicillin + kanamycin. 10 uL of phage dilutions in TN buffer were plated starting at a titre of 10^{-2} to 10^{-9} (labelled on plates as 1-8). The positive controls were 10 uL of plasmid containing ampicillin or kanamycin resistance genes diluted in TN buffer. The negative control was 10 uL of TN buffer.

References

22. Wong, S., Jimenez, S., and Slavcev, R.A. (2023). Construction and characterization of a novel miniaturized filamentous phagemid for targeted mammalian gene transfer. Microb. Cell Fact. 22, 124. https://doi.org/10.1186/s12934-023-02135-w.

68. Alting-Mees M.A., Short J.M. (1989). pBluescript II: gene mapping vectors. Nucleic Acids Res. 17, 9494. https://doi.org/10.1093/nar/17.22.9494.
